# Intracompartmental Pressure and Burn Depth Dynamics in Circumferential Deep Dermal Burns Treated with Bromelain-Based Enzymatic Debridement: A Retrospective, Hypothesis-Generating Single-Center Study

**DOI:** 10.3390/ebj7030047

**Published:** 2026-09-11

**Authors:** Dan Cristian Moraru, Mihai-Codrin Constantinescu, Alexandru-Hristo Amarandei, Dimitrie-Stefan Gologan, Stefana Avadanei-Luca, Andrei-Nicolae Gologan, Vladimir Poroch, Taher Hibi, Mihaela Pertea

**Affiliations:** 1 Faculty of Medicine, Grigore T. Popa University of Medicine and Pharmacy, 700115 Iași, Romania; cristian-dan.moraru@umfiasi.ro (D.C.M.); mihai-codrin.constantinescu@umfiasi.ro (M.-C.C.); mg-rom-33503@students.umfiasi.ro (D.-S.G.); stefana_luca@umfiasi.ro (S.A.-L.); andrei-nicolae.gologan@d.umfiasi.ro (A.-N.G.); vladimir.poroch@umfiasi.ro (V.P.); hibitaher95@gmail.com (T.H.); mihaela.pertea@umfiasi.ro (M.P.); 2 Department of Plastic Surgery, Reconstructive Microsurgery and Burn Unit, Sf. Spiridon Emergency County Hospital, 700111 Iași, Romania

**Keywords:** bromelain, NexoBrid^®^, intracompartmental pressure kinetics, EDT_50_, circumferential burns, laser Doppler imaging, burn-induced compartment syndrome, escharotomy, deep dermal burns, decompression

## Abstract

**Highlights:**

**What are the main findings?**

- Intracompartmental pressure (ICP) in circumferential deep burns declined progressively after bromelain-based enzymatic debridement, with no rebound and no clinical compartment syndrome.
- Decompression appeared slower in deeper burns, a pattern summarized by an exploratory descriptive parameter, the Enzymatic Decompression Half-Time (EDT_50_).

**What are the implications of the main findings?**

- The findings require prospective validation and do not replace surgical escharotomy when burn-induced compartment syndrome is established or when the burn lesion is not compatible with bromelain-based enzymatic debridement.
- Surgical escharotomy and bromelain-based enzymatic debridement are not competing strategies for the same indication; they are complementary instruments to be used in different operating conditions.

**Abstract:**

**Background/Objectives:** Circumferential deep dermal burns generate progressive sub-eschar pressure with risk of burn-induced compartment syndrome (BICS). Bromelain-based enzymatic debridement is a proposed non-surgical decompressive strategy, but the temporal kinetics of intracompartmental pressure (ICP) normalization and their relationship to burn depth are poorly characterized. We explored these kinetics and introduce, as an exploratory descriptive concept, the Enzymatic Decompression Half-Time (EDT_50_), mapping the ICP normalization curve stratified by Laser Doppler Imaging (LDI) burn depth. **Methods:** In this single-center retrospective study, 31 adults with circumferential deep dermal burns (IIb, TBSA ≤ 20%) treated with NexoBrid^®^ were analyzed. ICP was measured at five time points (T0–T24 h) and LDI at T0 and T24 h. Subgroup analyses were treated as descriptive because several subgroups were small. **Results:** ICP declined progressively with no rebound (repeated-measures ANOVA, *p* < 0.001). EDT_50_ appeared depth-dependent (high-flux 2.1 h; low-flux 5.9 h). No patient developed clinical BICS (0/31; 95% CI 0–11%). Pre-treatment LDI flux correlated negatively with ICP (r = −0.71) and EDT_50_ (r = −0.64). **Conclusions:** In this small cohort, enzymatic debridement was associated with progressive ICP normalization without clinical compartment syndrome. These hypothesis-generating findings require prospective validation; established BICS remains an absolute indication for surgical escharotomy.

## 1. Introduction

Circumferential deep thermal injuries of the extremities impose a pathophysiological burden qualitatively distinct from non-circumferential burns of equivalent depth. The rigid, desiccated necrotic eschar constrains the underlying compartment like a tourniquet, progressively preventing the outward expansion of edematous subcutaneous tissues during resuscitation [1,2]. The resulting sub-eschar pressure elevation compromises microvascular perfusion, impairs nerve conduction, and—if unrelieved—progresses to irreversible myonecrosis in what is collectively termed burn-induced compartment syndrome (BICS) [3].

The diagnosis of BICS rests on a combination of clinical assessment and objective pressure measurement. Clinically, it is defined by the progressive appearance of the six ‘P’ signs—pain disproportionate to injury, paresthesia, pallor, pulselessness, paralysis, and poikilothermia—although several of these are late and unreliable indicators of already-established ischemia [3]. Because clinical signs alone lack sensitivity in the obtunded, sedated, or regionally anesthetized patient, direct intracompartmental pressure (ICP) manometry is used as an objective adjunct. An absolute ICP above 30 mmHg has historically been regarded as the threshold for decompression, a value derived largely from the extremity trauma and tibial-fracture literature [4]. Increasingly, the perfusion gradient delta-P (diastolic blood pressure minus ICP) is considered physiologically more meaningful than absolute ICP, with delta-P below 30 mmHg indicating critical compromise of compartmental perfusion and a strong indication for surgical release [4]. These diagnostic thresholds frame the clinical decisions evaluated in the present study.

Surgical escharotomy is the historical standard for decompression: longitudinal incision releases the constraining eschar and immediately restores tissue compliance [1]. Its effectiveness is not in question; its limitations are. Escharotomy incisions are complicated by hemorrhage, iatrogenic injury to neurovascular structures, inability to achieve primary closure, obligatory split-thickness skin grafting, and—critically—formation of hypertrophic linear scar bands that may impair joint function and require secondary reconstruction [5,6]. These consequences are particularly significant in anatomically complex regions such as the hand, dorsal foot, and periarticular zones where scar contracture has long-term functional consequences.

Bromelain-based enzymatic debridement (NexoBrid^®^, MediWound Ltd.) dissolves denatured eschar collagen selectively, preserving viable dermis [7,8]. Its application in circumferential burns has been conceptualized as a ‘chemical escharotomy’: removal of the rigid constraining layer restores tissue compliance, allowing outward redistribution of sub-eschar fluid and reducing compartmental pressure [9,10]. Preclinical work by Krieger et al. reported ICP normalization within 4 h in an animal model of circumferential burn-induced compartment syndrome [11]. Subsequent clinical case series and retrospective data have supported clinical applicability [9,12]. Grünherz et al. reported that enzymatic debridement had largely replaced surgical escharotomy for circumferential extremity burns in a high-volume European center by 2021 [6].

A gap remains, however. Few clinical studies have systematically measured ICP at standardized time points during and after enzymatic debridement to characterize the temporal kinetics of pressure normalization. Current publications typically report results at a single post-treatment endpoint, do not address whether decompression is completed within or continues beyond the 4 h application window, and do not examine how the rate of normalization may depend on burn depth. This gap has potential clinical relevance: a clinician evaluating a patient at T4 h with ICP still elevated does not know whether to proceed to surgical escalation or whether post-application kinetics will deliver normalization. Furthermore, the relationship between burn depth—objectively quantifiable by Laser Doppler Imaging (LDI)—and ICP burden has not previously been well characterized.

Laser Doppler Imaging provides validated, contact-free quantification of dermal microvascular perfusion, with established thresholds predicting burn healing potential [13,14]. Perfusion flux correlates with residual dermal vascularity: high flux reflects superficial injury; low flux reflects deep dermal or full-thickness destruction [13,14]. We hypothesized that burn depth, reflected in LDI flux, is associated not only with baseline ICP but with the kinetics of enzymatic decompression—and examined this hypothesis by introducing, as an exploratory descriptive concept, the Enzymatic Decompression Half-Time (EDT_50_).

The present study’s aims were exploratory: (1) to describe the ICP normalization curve across five measurement points during and after enzymatic debridement; (2) to introduce and preliminarily evaluate the EDT_50_ concept and explore its possible depth-dependence; (3) to examine the correlations between LDI flux, ICP, and decompression kinetics; (4) to describe a preliminary ‘late normalization’ observation in deep-burn patients; and (5) to propose, as hypotheses for prospective testing, the clinical and paraclinical boundaries within which enzymatic debridement may be effective and safe as a decompressive strategy, explicitly distinguishing this from established BICS, where surgical escharotomy remains mandatory.

## 2. Materials and Methods

### 2.1. Study Design and Setting

This was a single-center retrospective study conducted at the Burn Unit of Sf. Spiridon Emergency County Hospital, Iași, Romania, over a 36-month period (January 2022–December 2024). Serial ICP and LDI assessments were part of the unit’s standardized monitoring protocol for circumferential deep dermal burns; the corresponding data were extracted retrospectively from the clinical records of consecutive patients meeting the eligibility criteria. Ethical approval was granted by the Institutional Review Board (Protocol Code 177/11.12.2025). All participants provided written informed consent for treatment and for the use of their data. The study was conducted in accordance with the Declaration of Helsinki, and reporting follows the STROBE statement for observational studies [15].

### 2.2. Inclusion and Exclusion Criteria

#### 2.2.1. Inclusion Criteria

(1) Age ≥ 18 years.
(2) Circumferential or near-circumferential (≥2/3 of limb circumference) thermal burn classified as deep dermal partial-thickness (IIb) by an experienced attending burn surgeon and confirmed by LDI.
(3) Upper or lower extremity burns (hand, forearm, arm, foot, or leg).
(4) Presentation within 12 h of burn injury.
(5) Absence of clinically established compartment syndrome (see exclusion criteria).
(6) Wound surface suitable for enzymatic debridement: moist eschar, or eschar suitable for pre-soaking hydration.
(7) TBSA ≤ 20%, in accordance with enzymatic debridement safety recommendations. The absolute minimum surface area was not restricted, provided the burn was circumferential/near-circumferential and posed a risk of compartment syndrome. The actual burn surface area treated with enzymatic debridement was specifically recorded to reflect true exposure.
(8) Intracompartmental pressure ≤ 40 mmHg at presentation.

#### 2.2.2. Exclusion Criteria

(1) Established BICS: ≥4 of 6 P-signs present (pain disproportionate to injury, paresthesia, pulselessness, pallor, paralysis, poikilothermia).
(2) Dominant full-thickness (grade III) burn pattern constituting >50% of the circumferential injury surface [16,17].
(3) ICP > 30 mmHg with ≥3 P-signs simultaneously present, or any sign of irreversible ischemia (absent distal pulse, fixed motor deficit).
(4) Chemical, high-voltage electrical (>1000 V), or blast burn injuries.
(5) Known hypersensitivity to bromelain, pineapple, or latex.
(6) Significant coagulopathy: Platelet count <50 × 10^9^/L, INR > 1.5, or therapeutic anticoagulation not safely bridgeable.
(7) Burn trauma-to-presentation interval > 12 h.
(8) Trunk burns (absent extremity-type compartments, different ICP physiology).
(9) Concurrent significant polytrauma requiring systemic stabilization as primary management priority.
(10) TBSA > 20%.

### 2.3. Pressure Subgroup Stratification

Based on the initial ICP measurement (T0) recorded at presentation, patients were classified into the following: Group I (ICP < 20 mmHg): sub-threshold; Group II (ICP 20–30 mmHg): warning zone per current guidelines [3,4]; Group III (ICP > 30 mmHg, ≤2 P-signs, delta-P [diastolic BP − ICP] > 30 mmHg, Doppler-confirmed distal perfusion): supra-threshold without established clinical BICS. Delta-P > 30 mmHg was verified in all Group III patients before enzymatic debridement was initiated, as delta-P is recognized as a more physiologically meaningful threshold than absolute ICP alone. Because the resulting subgroups were of unequal and, in the case of Group III, very small size (n = 3), subgroup contrasts were planned as descriptive rather than confirmatory analyses (see Section 2.8).

### 2.4. ICP Measurement Protocol

ICP measurements were performed with the Stryker STIC™ Intracompartmental Pressure Monitor System (Stryker Instruments, Kalamazoo, MI, USA) [18], a validated portable digital needle-manometry device. The side-port needle was introduced into the sub-eschar/subcutaneous compartment at the marked site after skin antisepsis; the system was zeroed to atmospheric pressure at the level of the measurement site before each reading, and a small bolus (<0.3 mL) of sterile saline was injected to establish fluid continuity before the equilibrium pressure was recorded. Five serial measurements were obtained per patient as part of the unit’s standardized monitoring schedule for circumferential burns (i.e., no study-specific procedure was added for research purposes):

- T0: Immediately before NexoBrid^®^ application (baseline);
- T2 h: Two h into application, accessed through a sterile port window in the occlusive dressing;
- T4 h: Immediately after gel removal at the end of 4 h application;
- T8 h: Eight h after debridement completion;
- T24 h: Twenty-four h after debridement completion.

At each time point, three readings were obtained at 1 min intervals, and the median was recorded.

In limbs exhibiting mixed-depth burns, the measurement site was meticulously chosen so that the side-port needle was introduced into the compartment directly underlying the deepest area of the burn (identified by the lowest pre-treatment LDI flux), as this region imposes the highest constrictive burden.

### 2.5. EDT_50_—Definition

EDT_50_ (Enzymatic Decompression Half-Time) is defined here as the elapsed time from T0 at which 50% of the total ICP reduction (T0 → T24 h) was achieved, calculated by linear interpolation between the two consecutive time points flanking the 50% threshold. A short EDT_50_ (<4 h) indicates decompression predominantly within the application window, whereas a long EDT_50_ (≥4 h) indicates post-application completion (‘post-enzymatic biomechanical creep’). Conceptually, EDT_50_ transposes the pharmacokinetic notion of a half-time (t½) to the mechanical domain, summarizing the speed of compartmental pressure normalization in a single, interpretable value. EDT_50_ is proposed here only as an exploratory, descriptive summary measure; it has not been validated as a clinical metric, and the present study should be regarded as its first, preliminary application to enzymatic debridement.

### 2.6. Laser Doppler Imaging Protocol

Burn depth was assessed using the Moor LDI2-BI (Moor Instruments Ltd., Axminster, UK) at T0 and T24 h. Scanning was performed in a temperature-controlled room (22–24 °C) after removal of topical agents and gentle cleansing, with the imaging head positioned perpendicular to and 20–30 cm from the wound surface; the low-power near-infrared laser raster-scans the surface, and the device computes a color-coded perfusion-flux map from the Doppler shift of moving erythrocytes, from which the mean flux of a standardized region of interest over the burn was extracted. Validated flux thresholds were applied: >400 PU (superficial, healing ≤ 14 days), 200–400 PU (deep dermal IIb, healing 14–21 days), <200 PU (deep/full-thickness, healing > 21 days) [13,14]. LDI depth stratification generated three subgroups: high flux (>300 PU, n = 10), mid flux (200–300 PU, n = 17), low flux (180–200 PU, n = 4). A representative clinical comparison of this color-coded output for high perfusion (superficial burn) versus low perfusion (deep dermal burn) is illustrated in Figure 1.

### 2.7. NexoBrid^®^ Application and Monitoring Protocol

NexoBrid^®^ (MediWound Ltd., Yavne, Israel) was applied per current European Consensus Guidelines [16,17]. Regional anesthesia was the preferred approach (brachial plexus or sciatic–femoral block). Systemic opioids and sedation were added as required. Pre-soaking with saline compresses (≥1 h) was performed when indicated. Enzymatic debridement was performed using 5 g vials of bromelain-enriched concentrate (mixed with 50 g of gel). Each 5 g vial suffices for treating roughly 450 cm^2^ of burn surface area (approximately 2.5% TBSA). These dosing calculations were strictly followed to ensure a uniform application layer 1.5–3 mm thick across the entire treated area. The gel was covered with Opsite^®^ (Smith & Nephew, Hull, UK) occlusive film, maintained for 4 h, then removed and irrigated with Prontosan^®^ (B. Braun Melsungen AG, Melsungen, Germany) (polyhexanide 0.1%). A predefined surgical escalation protocol required immediate escharotomy if ICP increased above T0, P-sign count progressed to ≥3, or distal perfusion deteriorated at any monitoring point. Clinical assessments were performed every 30 min during application and every 2 h through T24 h.

To minimize confounding effects on ICP kinetics, adjunctive conservative measures were strictly standardized across the cohort. All affected extremities were continuously elevated above heart level using standard positioning splints during both the application phase and the entire 24 h monitoring period. Furthermore, given the cohort’s limited burn extent (mean TBSA 9.4%, range 4–18%), fluid resuscitation was primarily enteral or restricted to baseline intravenous maintenance. This rigorous avoidance of over-resuscitation mitigated the risk of ‘fluid creep’, ensuring that systemic fluid shifts did not independently drive compartment hypertension.

### 2.8. Statistical Analysis

Statistical analysis used IBM SPSS Statistics, Version 28.0 (IBM Corp., Armonk, NY, USA) and GraphPad Prism, Version 10.0 (GraphPad Software, Boston, MA, USA). Serial ICP data for the whole cohort (n = 31) were analyzed by one-way repeated-measures ANOVA (Greenhouse–Geisser correction) with Bonferroni post hoc testing. Analyses stratified by pressure subgroup or by LDI depth subgroup—including the two-way mixed ANOVA (within-subjects factor: time; between-subjects factor: LDI depth subgroup) and all EDT_50_ subgroup contrasts—are reported descriptively and interpreted as exploratory and hypothesis-generating only. Several subgroups were small (Group III, n = 3; low-flux, n = 4) and cannot support robust inferential conclusions; the associated *p*-values are provided for completeness rather than as confirmatory evidence, and no major conclusion of this study rests on an inferential test performed within a subgroup of fewer than approximately ten patients. EDT_50_ was summarized descriptively per subgroup, with Kruskal–Wallis/Mann–Whitney statistics reported for transparency. Pearson correlations were computed on the whole cohort (n = 31) for (a) pre-treatment LDI flux vs. T0 ICP; (b) pre-treatment LDI flux vs. EDT_50_; (c) ΔFlux (T24 h − T0) vs. ΔP (T0 − T24 h). Effect sizes: η^2^ for ANOVA, r for correlations. Because no BICS event occurred, the corresponding risk estimate carries wide uncertainty (0/31; exact binomial 95% CI 0–11.2%), and the absence of events should not be interpreted as proof of efficacy. The significance threshold was *p* < 0.05; normality was assessed with the Shapiro–Wilk test.

## 3. Results

### 3.1. Cohort Characteristics

Thirty-one consecutive patients met the inclusion criteria and had a complete five-point ICP dataset. The cohort comprised 22 males (71.0%) and nine females (29.0%), with a mean age of 43.6 ± 14.2 years (range 19–71). Mean TBSA was 9.4 ± 4.8% (range 4–18%). In all cases, this reported TBSA corresponded entirely to the actual deep dermal surface area treated with enzymatic debridement on the affected extremities. Flame was the dominant mechanism (67.7%), followed by scald (25.8%) and contact (6.5%, resulting from prolonged dermal contact with hot solid objects, specifically industrial presses and domestic heating stoves in our cohort). Upper extremity burns accounted for 58.1%, lower extremity burns 35.5%, and bilateral extremity burns 6.5%. All burns were clinically classified as deep dermal (IIb), confirmed by LDI (mean pre-treatment flux 248 ± 62 PU). The ABSI score averaged 5.8 ± 2.1. Baseline characteristics by pressure subgroup are presented in Table 1; because Group III comprised only three patients, between-group differences in this table are descriptive.

### 3.2. Serial ICP Values: Full Time-Course

Table 2 presents serial ICP at T0 through T24 h for all subgroups. For the whole cohort, repeated-measures ANOVA showed a statistically significant main effect of time (F(4,120) = 147.3, *p* < 0.001, η^2^ = 0.83), indicating that a large proportion of total ICP variance was explained by the time factor. Bonferroni post hoc testing indicated differences between consecutive time point pairs (all *p* < 0.05), and no ICP rebound was observed between any consecutive measurements in any patient (Figure 2). An exploratory two-way mixed ANOVA suggested that the rate of ICP decline differed across LDI depth subgroups (time × depth-subgroup interaction F(8,112) = 7.9, *p* < 0.001, partial η^2^ = 0.35); given the small size of the depth subgroups, this result is regarded as consistent with, but not confirming, depth-dependence of the decompression kinetics and is interpreted as hypothesis-generating.

### 3.3. EDT_50_ Parameter: Depth-Stratified Decompression Kinetics

EDT_50_ appeared to stratify by LDI depth subgroup (Table 3; Figure 3), although the comparison involving the small low-flux subgroup (n = 4) is exploratory. High-flux patients (shallow IIb) had EDT_50_ = 2.1 ± 0.4 h, suggesting that most decompression occurred during the active application phase. Mid-flux patients had EDT_50_ = 3.8 ± 0.6 h, spanning the application and early post-application window. Low-flux patients (deep IIb approaching the full-thickness threshold) had EDT_50_ = 5.9 ± 0.8 h, suggesting that the majority of their decompression occurred post-application. EDT_50_ increased from 2.7 h (Group I) to 6.3 h (Group III) when stratified by ICP subgroup, a pattern consistent with convergent validity but limited by the small Group III size (Table 3, Figure 3).

### 3.4. Correlations: LDI Flux, ICP, and EDT_50_

Table 4 summarizes the three pre-specified whole-cohort correlations (Figure 4). The negative correlation between pre-treatment LDI flux and T0 ICP (r = −0.71) is consistent with deeper burns generating higher compartmental pressure at presentation. The negative correlation between LDI flux and EDT_50_ (r = −0.64) is consistent with deeper burns decompressing more slowly. The positive correlation between LDI flux change (ΔFlux) and ICP reduction (ΔP, r = +0.58) links the degree of enzymatic escharolysis to the magnitude of decompression, suggesting that more complete eschar removal was associated with greater ICP reduction.

### 3.5. Group III—A Preliminary ‘Late Normalization’ Observation in Supra-Threshold Patients

The three Group III patients (T0 ICP: 32, 35, and 38 mmHg, respectively) yield the study’s most clinically striking—though, given the small subgroup size (n = 3), necessarily preliminary, descriptive, and hypothesis-generating—observation. All had 2 P-signs (pain + paresthesia) and confirmed Doppler perfusion; delta-P values were 40, 37, and 34 mmHg. At T4 h, their ICP values were 26, 23, and 17 mmHg. At T8 h: 22, 20, and 17 mmHg. At T24 h: 22, 19, and 14 mmHg (group mean 18.3 ± 2.5 mmHg). The patient with T24 h ICP of 22 mmHg—still in the warning zone—was monitored through 48 h, at which point ICP was 18 mmHg with fully normalized clinical signs; no surgical escalation was required. In these three patients, the Group III EDT_50_ of 6.3 h corresponds to only about 37% of the eventual total ICP reduction having occurred by T4 h. Evaluating at T4 h alone might therefore misrepresent the trajectory of decompression; however, this inference is drawn from three patients only and requires confirmation in larger, prospective cohorts.

We describe this pattern, tentatively, as ‘late normalization’: a kinetic pattern in deep-burn patients in which the enzymatic application initiates escharolysis but much of the decompression appears to occur during the post-application phase, as biomechanical creep—viscoelastic stress dissipation within the newly mobilized soft-tissue compartment—continues over 8–24 h. In these cases, the pattern did not reflect inadequate treatment; it suggests that the clinically relevant monitoring window may extend beyond T4 h in deeper burns. This remains a hypothesis to be tested rather than an established phenomenon.

### 3.6. Clinical Outcomes and Wound Healing

The primary safety endpoint—clinical BICS development—was not observed in any patient (0/31; exact 95% CI 0–11.2%); no patient required escalation to surgical escharotomy. Given the sample size, this absence of events indicates that clinical BICS was uncommon under this monitored protocol but does not, by itself, establish efficacy. Table 5 summarizes clinical outcomes.

### 3.7. LDI Wound Assessment and Post-Treatment Findings

Post-treatment LDI at T24 h showed an overall increase in mean flux from 248 ± 62 to 312 ± 71 PU (mean increase 64 ± 38 PU, +26.1%), consistent with removal of the low-perfusion eschar layer and exposure of viable dermis. Flux > 200 PU (viable dermis) was recorded in 28/31 patients (90.3%). In three patients (9.7%), post-treatment flux remained <200 PU, indicating deeper residual injury; all three required surgical skin grafting and were re-classified as borderline IIb/III. The positive correlation between ΔFlux and ΔP (r = +0.58) is consistent with a mechanistic link between escharolysis completeness and ICP reduction magnitude.

Clinically, eschar structure disruption was macroscopically evident at T4 h upon dressing removal, manifesting as softening, liquefaction, and easy mechanical wiping of the necrotic layer, thereby physically restoring cutaneous pliability.

### 3.8. Representative Clinical Cases

Representative clinical cases illustrating the enzymatic debridement sequence are presented in Figure 5, Figure 6, Figure 7 and Figure 8. No cases of surgical escharotomy (incisional release) are included; all illustrated patients were managed with enzymatic debridement exclusively.

## 4. Discussion

### 4.1. Central Findings and Their Clinical Context

This retrospective study reports several observations that add to the enzymatic debridement literature but that should be read as preliminary and hypothesis-generating given its single-center, retrospective design and modest sample size. First, ICP declined progressively across all five measurement points and in all patient subgroups, with no rebound documented at any interval. This is mechanistically reassuring and consistent with a stable, directionally sustained decompressive effect of enzymatic escharolysis: we observed no oscillatory ‘pressure bounce’ following disruption of eschar architecture, as might theoretically occur if removal of the mechanical constraint were followed by reactive edema formation faster than drainage. The absence of rebound across 31 patients and 155 measurement events is consistent with a unidirectional biomechanical consequence of escharolysis rather than pressure redistribution, although a single-arm design cannot exclude it definitively.

Second, and more novel, is the apparent kinetic structure of decompression. The EDT_50_ concept—applied here, to our knowledge, for the first time in the enzymatic debridement context—suggests that decompression may not be a step-function completed within the application window but a time-extended process whose speed appears to be related to burn depth. The finding that EDT_50_ in low-flux (deep IIb) patients was 5.9 ± 0.8 h would imply that, at T4 h, the decompressive process in a low-flux patient is still less than halfway complete. This raises a previously unframed hypothesis: that the T4 h assessment may not be the appropriate endpoint for evaluating enzymatic decompression adequacy in deep burns, and that the monitoring window might need to extend to T8 h or T24 h, individualized by LDI flux. This possibility requires prospective testing before it can inform practice.

Third, the correlations between LDI flux, ICP, and EDT_50_ may link two independently validated clinical tools—LDI and compartment-pressure manometry. That deeper burns were associated with higher baseline compartmental pressure (r = −0.71) is physiologically plausible: deeper denaturation produces a more consolidated, less hydrated, more rigid eschar, imposing greater constraint on the underlying compartment. That burn depth also correlated with decompression speed (r = −0.64 with EDT_50_) is the novel, exploratory contribution: deeper eschar may require more time for enzymatic proteolysis to progress to the viable-dermis boundary, delaying restoration of sufficient compliance to relieve pressure.

### 4.2. A Proposed Mechanism: Post-Enzymatic Biomechanical Creep

The continued ICP decline between T4 h and T24 h—observed particularly in low-flux and Group III patients—is what we describe as ‘post-enzymatic biomechanical creep.’ The term borrows from biomechanical materials science, where ‘creep’ describes the time-dependent deformation of a viscoelastic material under sustained load [19]. Under this proposed model, this progressive pressure decline is physiologically supported by the macroscopic liquefaction of the eschar. Bromelain selectively cleaves denatured collagen, destroying the rigid tridimensional matrix of the eschar [7]. The immediate restoration of cutaneous pliability removes the primary mechanical constricting element at T4 h (the tourniquet effect), but the underlying soft tissue—edematous, mechanically stressed, with disrupted extracellular matrix architecture—requires additional time to redistribute outward. The process would be analogous to the response of viscoelastic tissue to sudden release of constraint: stress is not instantaneously dissipated but decays over time as fluid redistributes and the extracellular matrix relaxes. This mechanistic account is a hypothesis consistent with the observed kinetics rather than a demonstrated pathway.

Supporting this interpretation is the observation that the cumulative % ICP reduction at T4 h was substantially lower in low-flux patients (≈32%) than in high-flux patients (≈75%), despite both groups ultimately reaching comparable absolute ICP by T24 h. The application phase appears to initiate decompression, and the post-application phase to complete it. The clinical implication we propose—for prospective evaluation—is that enzymatic debridement might be conceptually framed not as a 4 h procedure but as a 24 h decompressive process in which the active application phase is the first and most visible, but not necessarily the concluding, step.

### 4.3. Group III: Late Normalization and the Decision Not to Escalate

The Group III subgroup—three patients with T0 ICP > 30 mmHg and 2/6 P-signs—represents the highest-stakes clinical scenario in this study. The conventional teaching of ICP > 30 mmHg as an absolute threshold for surgical decompression derives primarily from the trauma fasciotomy literature and was not established specifically for burns [3]. The delta-P concept—subtracting ICP from diastolic blood pressure to estimate the microvascular perfusion-pressure gradient—has been argued to be physiologically superior to absolute ICP alone [4]. All three Group III patients had delta-P > 30 mmHg (34–40 mmHg), indicating maintained perfusion pressure despite absolute ICP exceeding threshold, which supported the monitored enzymatic approach in these specific, closely observed cases.

At T4 h, mean Group III ICP was 22.0 mmHg—above the 20 mmHg warning threshold and above the surgical threshold already exceeded at T0. Without the kinetics data, this T4 h reading would reasonably prompt consideration of surgical escalation. With the kinetics data, and with maintained perfusion and absent P-sign progression, the observed trajectory through T8 h (19.8 mmHg) to T24 h (18.3 mmHg) is consistent with late but complete enzymatic decompression in these three patients. These observations generate the hypothesis that an EDT_50_-informed monitoring schedule—assessing a deep, low-flux patient at T8 h–T24 h rather than at T4 h—might reduce unnecessary escalation. The clinical significance of this “delayed normalization” is paramount: it suggests that in deep burns, a borderline ICP at T4 h does not automatically represent treatment failure. If the microvascular perfusion (delta-P) is maintained, avoiding premature surgical escalation and continuing monitoring can safely allow the biomechanical creep to finalize the decompression.

These three cases must be contextualized strictly. The protocol required the following g: (a) delta-P > 30 mmHg confirmed before enzymatic treatment; (b) ≤2 P-signs; (c) continuous-wave Doppler-confirmed perfusion; (d) monitoring every 30 min during application and every 2 h post-treatment; (e) immediate surgical escalation available. These conditions are not negotiable. The supra-threshold ICP group should not be interpreted as expanding the enzymatic decompression indication to patients with established BICS. Any loss of Doppler flow, progression to ≥3 P-signs, or any uncertainty about perfusion adequacy is an absolute criterion for immediate surgical escharotomy.

### 4.4. Surgical Escharotomy: Its Preserved Primacy and Complementary Role

This study is deliberately framed so as not to diminish the role of surgical escharotomy. If anything, by attempting to describe the window in which enzymatic debridement may be effective, it also delineates the clinical space where surgery remains the only appropriate choice. Surgical escharotomy offers immediate, verifiable mechanical decompression—a property enzymatic debridement does not share. The surgeon makes an incision, sees and feels the tissue separate, and can immediately confirm decompression. This predictability is its paramount advantage in time-critical scenarios.

The literature consistently documents the limitations of enzymatic debridement in specific contexts. Grünherz et al. reported that surgical escharotomy remained the dominant approach in patients with larger burns (mean TBSA 47%) and a higher proportion of grade III injury [6,10]. Tapking et al. found enzymatic debridement to be significantly less effective in scalds than in flame burns, attributed to delayed zone-of-stasis progression and altered eschar characteristics [20]. The European Consensus Guidelines explicitly restrict enzymatic debridement in full-thickness burns to anatomically demanding regions and reserve surgical approaches for dominant grade III circumferential patterns [16,17]. The present study’s inclusion of only grade IIb burns and exclusion of scald-dominant supra-threshold cases is consistent with these boundaries.

The appropriate clinical framework is therefore complementarity, not competition. Enzymatic debridement may occupy a defined physiological window: deep dermal circumferential burns with LDI-confirmed IIb depth, ICP in the warning zone or supra-threshold range with maintained delta-P, thermal mechanism, and early presentation with full monitoring capacity. Surgical escharotomy remains the standard—immediate, reliable, and non-negotiable—outside this window and should never be delayed when any ambiguity exists about the adequacy of enzymatic decompression or when monitoring conditions cannot support the extended post-application surveillance that deep-burn enzymatic decompression appears to require.

### 4.5. LDI as a Potential Dual-Purpose Tool: Depth Prediction and Kinetic Forecasting

The established role of LDI in burn depth assessment and healing prediction is well supported [13,14,21]. The present data extend this utility by suggesting that pre-treatment LDI flux is associated with baseline ICP burden (r = −0.71) and, more importantly, with decompression kinetics (r = −0.64 with EDT_50_). This means that a single admission LDI scan—already routine in most European burn centers—could simultaneously inform three decisions: wound healing prognosis; expected ICP at presentation; and the likely duration of post-debridement monitoring required. Integrating these outputs from a single non-invasive measurement may represent a clinical efficiency gain.

Post-treatment LDI at T24 h may add a fourth decision input: wound-management strategy after escharolysis. Deep second-degree burns are notoriously susceptible to early progressive deepening due to ischemia in the zone of stasis [22]. The finding that 90.3% of patients had T24 h flux > 200 PU confirms the selectivity of enzymatic debridement in preserving dermal microvascular perfusion [7,8]. However, it is crucial to distinguish between microvascular preservation and true ‘healing potential’. Preserving blood flow in the zone of stasis mitigates ischemic progressive deepening, but if the initial thermal insult has thoroughly destroyed the deep epithelial appendages, the wound will still require surgical grafting despite an adequately perfused vascular bed. The 9.7% of patients with residual flux < 200 PU represent either initial clinical underestimation or true ischemic depth conversion. Post-treatment LDI directed these specific low-flux patients to early surgical management, while acknowledging that even high-flux patients must be monitored for actual epithelialization capacity. This is consistent with a role for T24 h LDI as a protocol-embedded decision tool rather than an absolute predictor of spontaneous healing.

### 4.6. An Exploratory Framework for Hypothesis Testing

Drawing on the present preliminary data and existing evidence [4,6,12,16,18,22], Table 6 and Figure 9 outline an exploratory framework that integrates ICP, LDI flux, clinical signs, and EDT_50_-informed monitoring duration. Its purpose is to make the study’s hypotheses explicit and testable, so that they can be prospectively evaluated before any clinical use.

### 4.7. Comparison with Published Literature

The mechanistic evidence base for enzymatic escharolysis as a decompressive modality rests primarily on Krieger et al.’s animal model [11], which reported ICP normalization within the 4 h treatment window. The present data are consistent with this finding for superficial IIb burns (high-flux EDT_50_ ≈ 2 h) but suggest that deep IIb burns may require post-application monitoring to T24 h. Fischer et al. reported that enzymatic debridement prevented operative escharotomy in circumferential distal upper-extremity burns [9], and Cordts et al. reported its feasibility even in severely burned upper extremities [23], but neither study included ICP measurements. The retrospective series by Grünherz et al. [6] most closely relates to our work but addressed a comparison population (surgical vs. enzymatic) rather than characterizing kinetics and did not include LDI data. The systematic review by Shoham et al. summarized healing and surgical-outcome benefits but did not address ICP dynamics [24]. The present study therefore addresses a specific and previously under-examined niche in the evidence landscape.

Notably, the Ziegler et al. state-of-the-art review [25] identified the need for studies with objective measurements to help validate enzymatic decompression as a clinical standard. The present data contribute a preliminary five-point time-series of ICP with concurrent LDI assessment. The correlation structure identified here—particularly the LDI flux–EDT_50_ relationship—is a novel exploratory observation that was not inferable from previously published datasets, though it requires confirmation. Taken together, these objective mechanistic observations provide a preliminary pathophysiological rationale consistent with the overall clinical utility of the bromelain-enriched enzyme mixture (NexoBrid^®^) previously reported by Pertea et al. [26].

## 5. Limitations

This study has important limitations that constrain the strength of its conclusions. (1) It is a single-center, retrospective, single-arm study of only 31 patients; several subgroups are very small—most notably Group III (n = 3) and the low-flux depth subgroup (n = 4). Inferential statistics performed within such small subgroups cannot provide robust evidence, and all subgroup contrasts (including the two-way mixed ANOVA and subgroup EDT_50_ comparisons) are therefore reported as descriptive and hypothesis-generating; the study’s principal novel constructs—the EDT_50_ parameter, the ‘late normalization’ pattern, and the exploratory framework of Section 4.6—rest substantially on these limited observations and must be regarded as preliminary rather than validated. (2) The absence of any BICS event (0/31) carries wide statistical uncertainty (exact 95% CI 0–11.2%) and does not establish efficacy. (3) ICP was measured at the sub-eschar/subcutaneous level rather than the deep fascial compartment; subfascial measurement was available as an escalation tool but not performed systematically. (4) EDT_50_ is calculated by linear interpolation between five time points, which may introduce estimation error if the true curve is non-linear between points. (5) LDI in the first hours post-injury may underestimate final burn depth owing to incomplete zone-of-stasis declaration [20]. (6) Longer-term wound healing, scar quality, and functional outcome data were secondary and exploratory. (7) The retrospective, single-arm design carries inherent risks of selection and information bias and precludes direct quantitative comparison with surgical escharotomy kinetics—risks mitigated, but not eliminated, by consecutive sampling and reliance on a pre-existing standardized measurement protocol. Prospective, adequately powered, multi-center studies are required before any of the proposed constructs or frameworks could be considered for clinical use.

## 6. Conclusions

This study provides a preliminary serial-measurement characterization of ICP normalization kinetics during and after enzymatic debridement in circumferential deep dermal burns, stratified by objective LDI-based depth assessment. In this small retrospective cohort, enzymatic debridement was associated with progressive, sustained ICP reduction and no clinical BICS (0/31; 95% CI 0–11%). The kinetics appeared depth-dependent: shallow IIb burns tended to decompress rapidly during the application phase (EDT_50_ ≈ 2 h), whereas deep IIb burns appeared to continue decompressing post-application (EDT_50_ ≈ 6 h). No ICP rebound was observed. The preliminary ‘late-normalization’ pattern seen in the three Group III patients raises the hypothesis that T4 h may represent a midpoint rather than an endpoint of decompression in deep burns, with potential implications for when decompression adequacy is assessed—implications that require prospective testing.

The EDT_50_ parameter, associated with pre-treatment LDI flux (r = −0.64), is proposed as an exploratory concept that, if validated prospectively, might help individualize post-debridement monitoring duration; it is not yet a clinically actionable tool. Similarly, the integration of LDI depth data with ICP manometry is presented as a candidate dual-instrument approach for future evaluation rather than an established framework.

On the basis of these data, enzymatic debridement appears to be a promising decompressive strategy within a narrowly defined boundary: circumferential deep dermal burns, preserved distal Doppler perfusion, delta-P > 30 mmHg, <3 clinical P-signs, thermal mechanism, and full monitoring capacity with immediate surgical escalation available. Within this boundary, it may achieve decompression while preserving dermis, avoiding incisional morbidity, and enabling early rehabilitation. Outside this boundary, surgical escharotomy is the safest, most reliable, and only appropriate intervention. Established BICS with ≥4 P-signs, absent distal pulse, irreversible ischemia, dominant grade III burns, scald mechanism with delayed zone-of-stasis development, TBSA > 20%, or absence of monitoring infrastructure are all absolute contraindications to enzymatic debridement as primary decompression. These modalities are complementary instruments for distinctly different operating conditions, not competing strategies for the same indication. All of the above conclusions are hypothesis-generating and require prospective, multi-center validation before informing clinical practice.

## Figures and Tables

**Figure 1:**
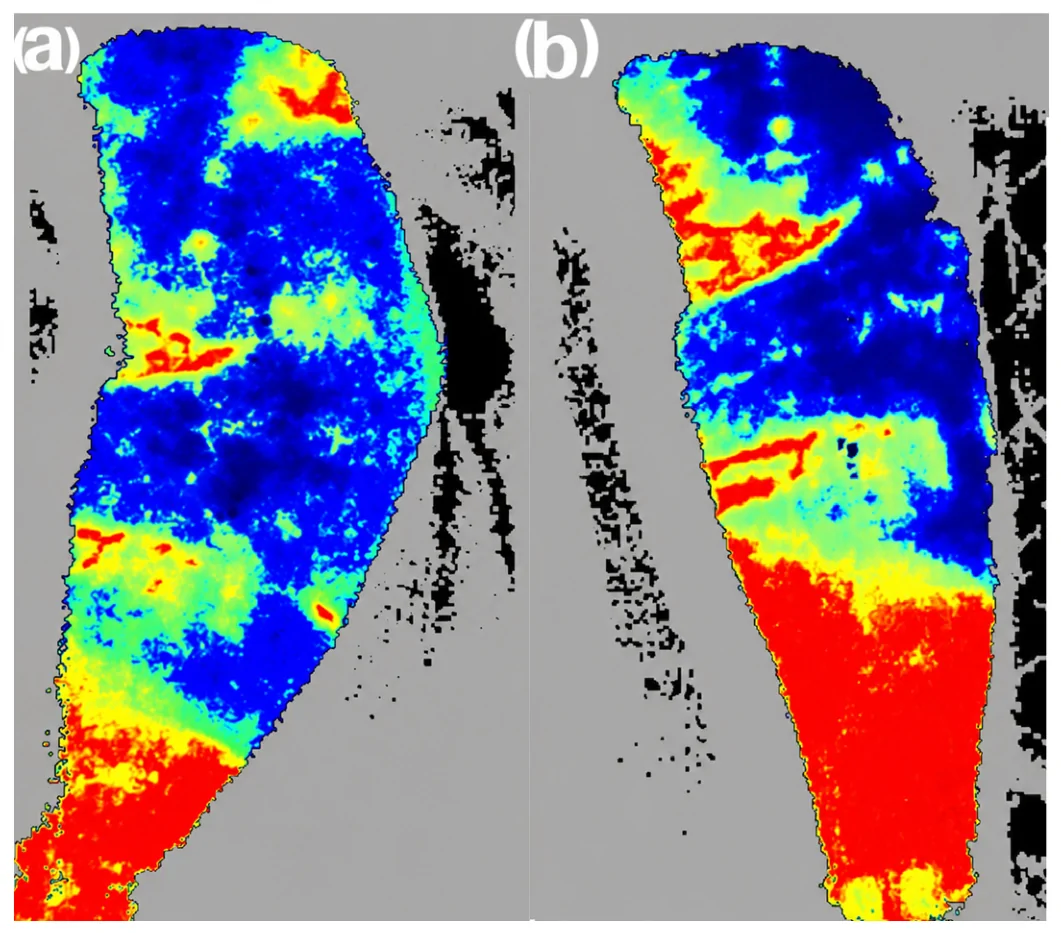
Burn depth assessment using Laser Doppler Imaging (LDI) to map skin blood perfusion. (**a**) The left section displays a deep dermal burn where the LDI scan is dominated by cool colors (blue and green), signifying severely reduced or absent blood perfusion due to destruction of the capillary network. (**b**) The right section displays a mixed-depth burn; the distal portion (about half of the scanned area) exhibits a superficial dermal injury characterized by high blood flow, indicated by warm colors (predominantly red).

**Figure 2:**
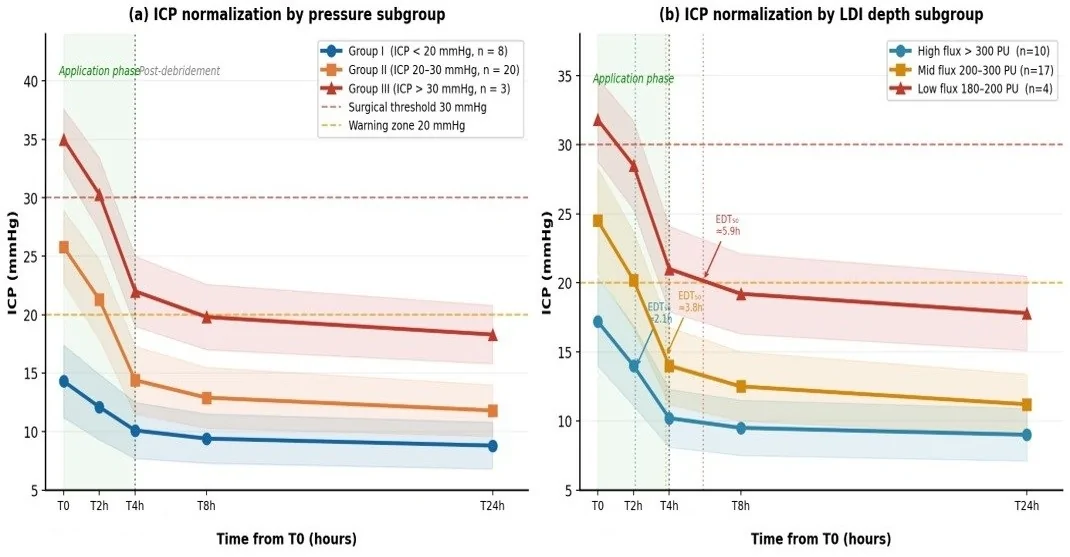
Serial ICP normalization curves during and after enzymatic debridement across five time points (T0, T2 h, T4 h, T8 h, T24 h). (**a**) Stratification by ICP subgroup (Group I: blue; Group II: orange; Group III: red). (**b**) Stratification by pre-treatment LDI depth subgroup (high flux > 300 PU: teal; mid flux 200–300 PU: amber; low flux 180–200 PU: red). The vertical dotted line marks the end of enzymatic application (T4 h). Shaded bands represent ±1 SD. Note the continued ICP decline from T4 h to T24 h in the low-flux and Group III subgroups (post-enzymatic biomechanical creep). Horizontal dashed lines: 30 mmHg (surgical threshold) and 20 mmHg (warning zone). Curves for the smallest subgroups (Group III, n = 3; low flux, n = 4) are descriptive.

**Figure 3:**
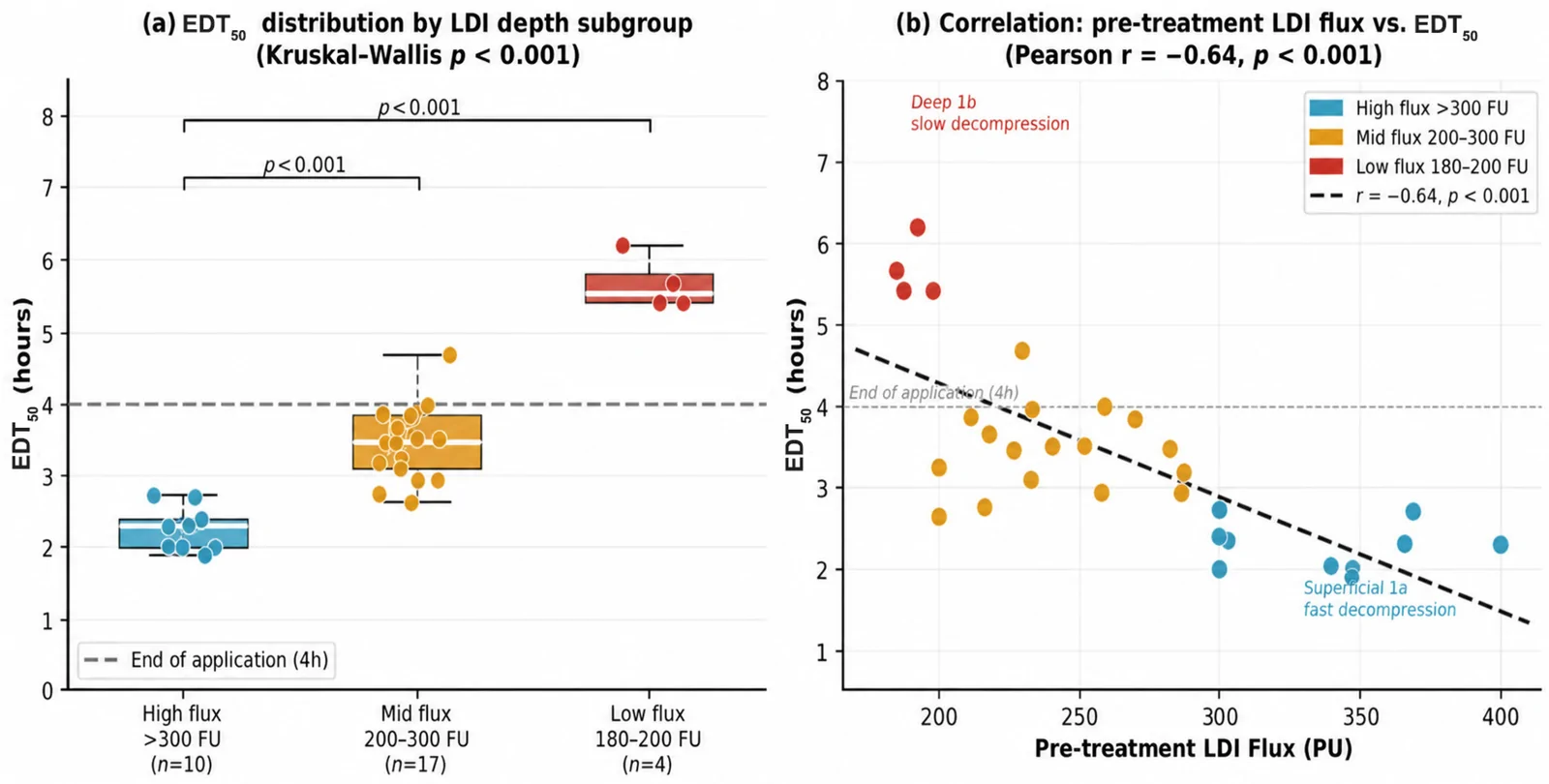
EDT_50_ distribution and its relationship with LDI depth. (**a**) Box-and-whisker plot of EDT_50_ by LDI depth subgroup with individual data points (jittered). Horizontal dashed line at 4 h = end of application. Significance brackets from Kruskal–Wallis Dunn’s post hoc are shown for transparency; contrasts involving the low-flux subgroup (n = 4) are exploratory. (**b**) Scatter plot of pre-treatment LDI flux vs. EDT_50_ across all patients (n = 31), color-coded by LDI subgroup. Dashed line: linear regression; Pearson r = −0.64 (*p* < 0.001). Deeper burns (lower flux) tended to have longer EDT_50_.

**Figure 4:**
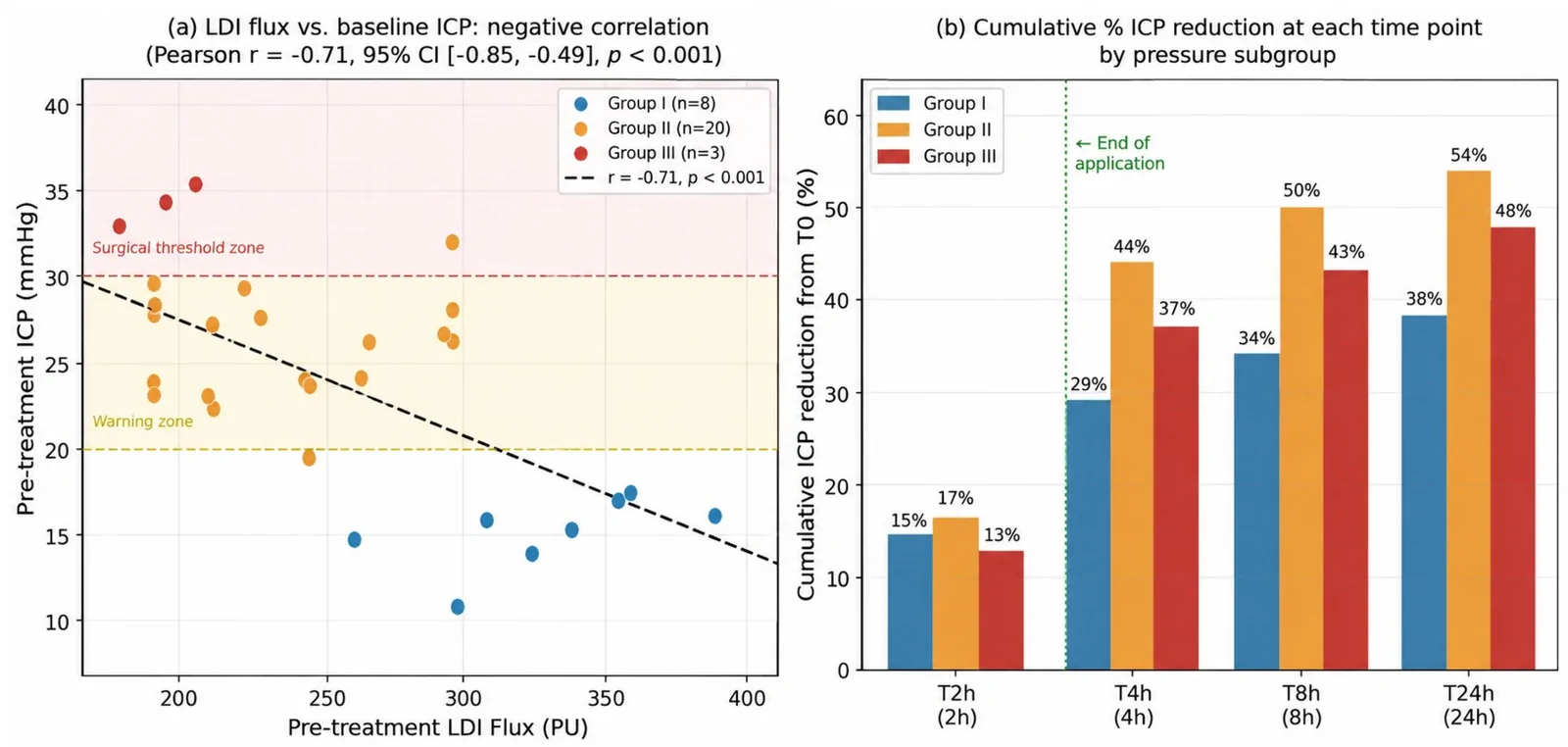
LDI–ICP correlation analysis. (**a**) Scatter plot of pre-treatment LDI flux (PU) vs. T0 ICP (mmHg). Points color-coded by pressure subgroup. Dashed regression line; r = −0.71 (*p* < 0.001). Shaded zones: yellow = warning zone (20–30 mmHg), red = surgical threshold zone (>30 mmHg). (**b**) Grouped bar chart of cumulative % ICP reduction at each measurement time point by pressure subgroup. The vertical dotted line separates the application phase (T4 h) from the post-debridement phase.

**Figure 5:**
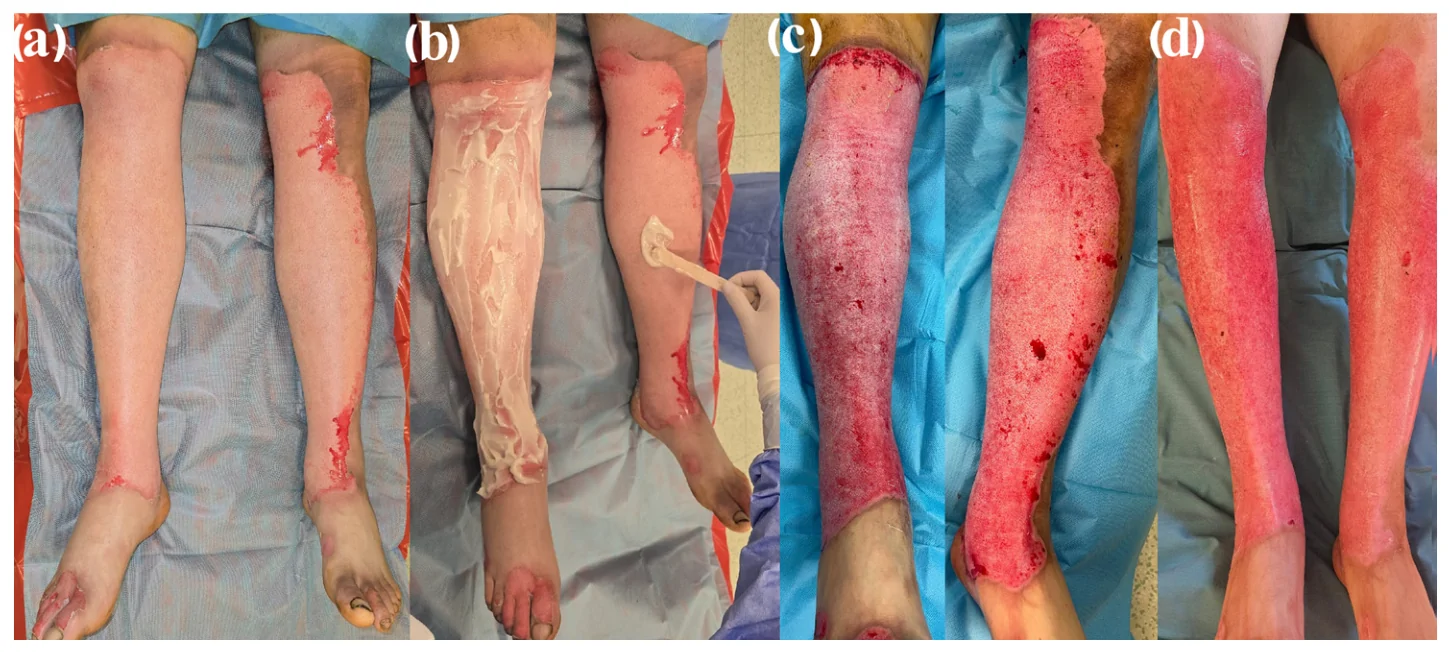
Circumferential deep dermal (IIb) burn of the lower extremity managed with enzymatic debridement. (**a**) Pre-treatment: Circumferential eschar with distal edema; T0 ICP = 24 mmHg (Group II). Pre-treatment LDI T0 flux was 285 PU. (**b**) NexoBrid^®^ gel application under occlusive dressing; wound irrigation after gel removal at 4 h; T4 h ICP = 14 mmHg (−42%). (**c**) Clinical appearance at T24 h (bilateral lower extremities) demonstrating early viable wound beds; T24 h ICP = 12 mmHg. (**d**) Day 14: advanced re-epithelialization approaching wound closure; no skin grafting required. LDI T24 h flux 335 PU (high-flux conversion, viable dermis confirmed).

**Figure 6:**
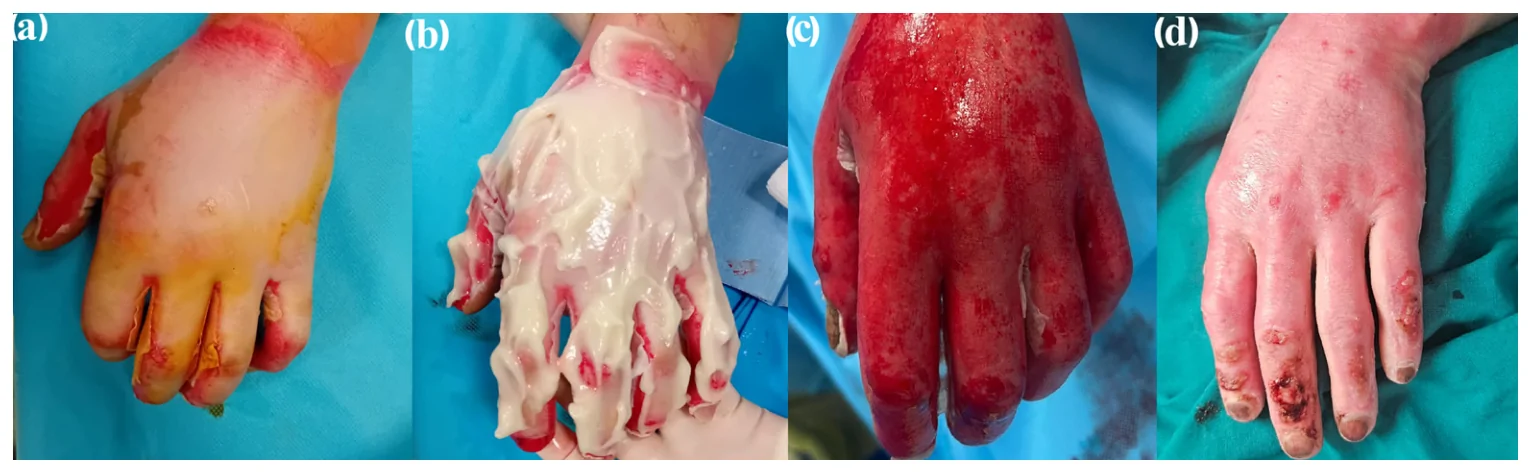
Circumferential deep dermal hand burn (IIb, Group II). (**a**) Pre-treatment: Circumferential eschar with digital edema and tension; ICP T0 = 26 mmHg; 2 P-signs. (**b**) NexoBrid^®^ gel applied under occlusive dressing. (**c**) Immediate post-debridement at T4 h: Pink viable wound bed; ICP T4 h = 15 mmHg (−42%); EDT_50_ ≈ 2.4 h (high-flux pattern). (**d**) Day 14 follow-up: Advanced healing without surgical release incisions; preserved hand architecture.

**Figure 7:**
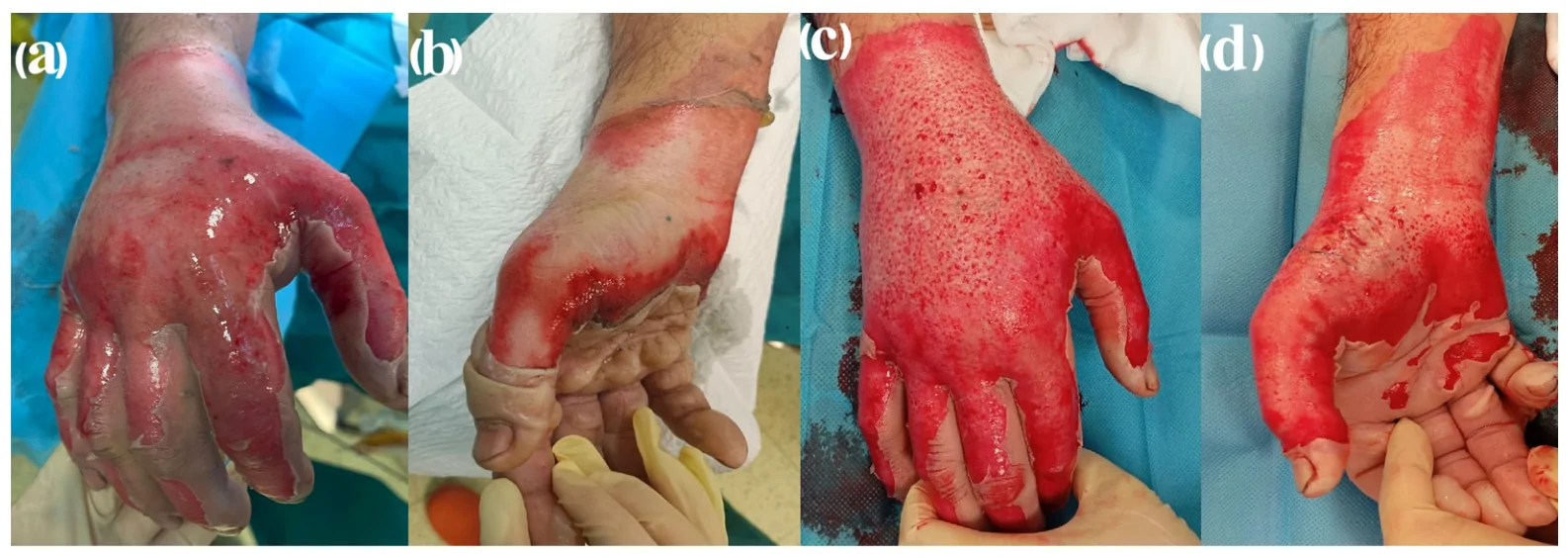
Circumferential deep dermal burns of the hand and wrist with proximal extension (Group II, ICP T0 = 28 mmHg). (**a**,**b**) Pre-treatment views demonstrating circumferential eschar and digital compression. (**c**,**d**) Post-debridement appearance: Eschar removed with preservation of digit architecture and tissue planes; no incisional scarring. ICP T24 h = 13 mmHg. Wound managed conservatively to healing without grafting.

**Figure 8:**
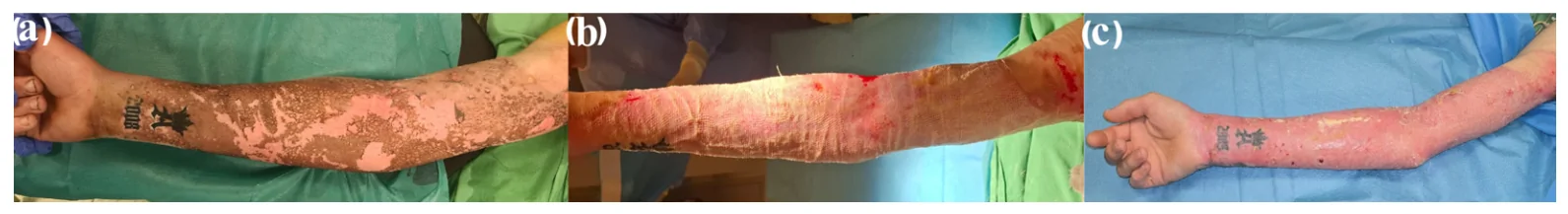
Circumferential deep dermal (IIb) burn of the forearm (upper extremity, Group II). (**a**) Pre-treatment: Circumferential eschar generating compartmental tension; ICP T0 = 25 mmHg; 1 P-sign. (**b**) Post-debridement Day 3: This panel illustrates the characteristic formation of a post-enzymatic pseudoeschar (a fibrinous exudate layer) following NexoBrid^®^ application, which can temporarily alter the macroscopic shape and apparent circumference of the limb. Beneath this transient layer, the time point confirms the definitive viability of the pink wound bed and the onset of early spontaneous re-epithelialization, demonstrating the absence of progressive ischemia in the zone of stasis. Decompression was achieved rapidly within the application window (ICP T4 h = 14 mmHg, −44% reduction; EDT_50_ = 2.1 h), corresponding to a high-flux LDI pattern. (**c**) Day 14 follow-up: advanced re-epithelialization approaching complete wound closure.

**Figure 9:**
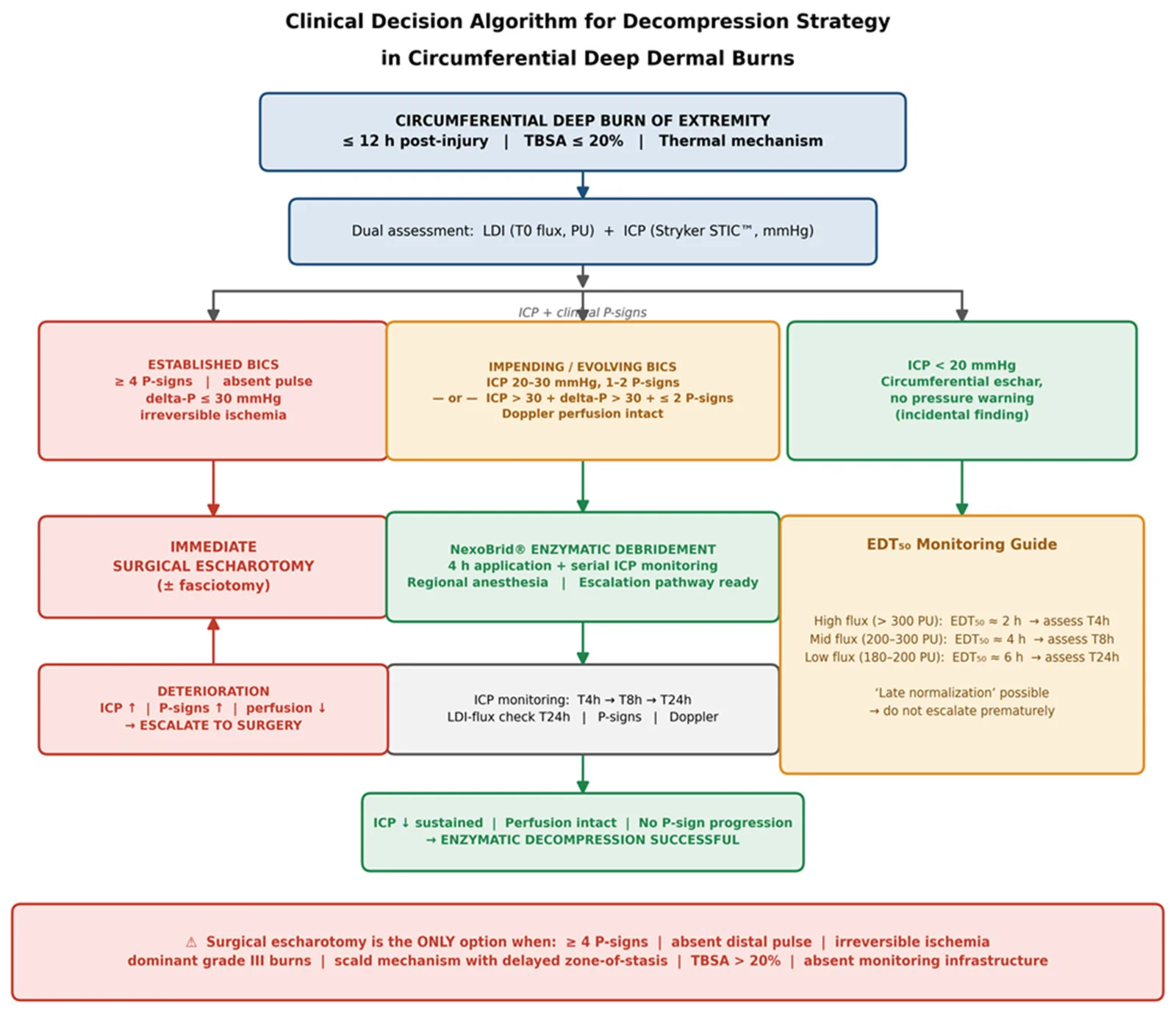
Exploratory decision scheme (hypothesis-generating; not a validated clinical algorithm) for decompression strategy in circumferential deep dermal burns, integrating ICP measurement, clinical P-sign assessment, delta-P calculation, and LDI depth subgroup to distinguish candidate enzymatic vs. surgical pathways and to frame hypotheses about individualized post-debridement monitoring duration using the EDT_50_ concept. Surgical escharotomy indications are highlighted in red; candidate enzymatic pathways in green; the EDT_50_ monitoring panel (amber) states hypotheses for prospective testing, not recommendations. Any clinical deterioration during monitoring immediately triggers surgical escalation regardless of ICP trajectory. This scheme requires prospective validation before any clinical use.

**Table 1:** Baseline demographic and clinical characteristics by ICP subgroup. Values are mean ± SD unless stated. Group III (n = 3) values are descriptive.

Characteristic	Group I (ICP < 20, n = 8)	Group II (ICP 20–30, n = 20)	Group III (ICP > 30, n = 3)	All Patients (n = 31)
Age (years)	41.3 ± 12.1	44.2 ± 15.0	42.0 ± 11.5	43.6 ± 14.2
Male sex, n (%)	6 (75%)	14 (70%)	2 (67%)	22 (71%)
TBSA (%)	7.2 ± 3.1	9.8 ± 5.0	12.3 ± 2.9	9.4 ± 4.8
ABSI score	4.9 ± 1.6	5.9 ± 2.2	7.3 ± 1.5	5.8 ± 2.1
Mechanism: Flame, n (%)	4 (50%)	14 (70%)	3 (100%)	21 (68%)
Mechanism: Scald, n (%)	3 (38%)	5 (25%)	0 (0%)	8 (26%)
Upper extremity, n (%)	5 (63%)	12 (60%)	1 (33%)	18 (58%)
Lower extremity, n (%)	3 (37%)	7 (35%)	1 (33%)	11 (36%)
T0 ICP (mmHg)	14.3 ± 3.1	25.8 ± 3.1	35.0 ± 2.6	23.1 ± 6.4
Pre-Tx LDI flux (PU)	312 ± 41	235 ± 55	188 ± 28	248 ± 62
P-signs at admission	0–1 (all)	1–2	2 (all)	—
Delta-P (dBP − ICP, mmHg)	≥50 (all)	38–52	34–40	—
Time injury → treatment (h)	3.2 ± 1.4	3.9 ± 2.0	4.1 ± 1.7	3.8 ± 1.9

ABSI: Abbreviated Burn Severity Index; TBSA: total body surface area; ICP: intracompartmental pressure; LDI: Laser Doppler Imaging; PU: perfusion units; dBP: diastolic blood pressure; Pre-Tx: pre-treatment; n: number; h: hour.

**Table 2:** Serial ICP values (mean ± SD, mmHg) at five measurement points. Values in parentheses = % reduction relative to T0. Group III (n = 3) values are descriptive.

Subgroup	T0 (Pre)	T2 h (Mid-Appl.)	T4 h (End Appl.)	T8 h	T24 h	% Red. T24 h
Group I (n = 8)	14.3 ± 3.1	12.1 ± 2.8 (−15%)	10.1 ± 2.4 (−29%)	9.4 ± 2.1 (−34%)	8.8 ± 2.0	−38.5%
Group II (n = 20)	25.8 ± 3.1	21.3 ± 3.4 (−17%)	14.4 ± 2.9 (−44%)	12.9 ± 2.6 (−50%)	11.8 ± 2.2	−54.3%
Group III (n = 3)	35.0 ± 2.6	30.3 ± 3.1 (−13%)	22.0 ± 3.0 (−37%)	19.8 ± 2.8 (−43%)	18.3 ± 2.5	−47.7%
All patients (n = 31)	23.1 ± 6.4	19.3 ± 5.8 (−16%)	13.9 ± 4.2 (−40%)	12.4 ± 3.9 (−46%)	11.6 ± 3.5	−49.8%

n: number. All pairwise consecutive time point differences in the whole cohort were significant (Bonferroni, *p* < 0.05). No ICP rebound occurred at any time point in any patient. In Group III, mean ICP at T4 h (22.0 mmHg) remained above the 20 mmHg warning threshold and continued to fall to 18.3 mmHg at T24 h without clinical deterioration (‘late normalization’; see Section 3.5).

**Table 3:** EDT_50_ (mean ± SD, hours) by ICP subgroup and by LDI flux depth subgroup.

Stratification	Subgroup	n	EDT_50_ Mean ± SD (h)	Comparison *p*-Value
By ICP subgroup	Group I (ICP < 20 mmHg)	8	2.7 ± 0.5	Reference
	Group II (ICP 20–30 mmHg)	20	4.1 ± 0.7	0.001 vs. Group I
	Group III (ICP > 30 mmHg)	3	6.3 ± 0.6	<0.001 vs. Group I (exploratory)
By LDI depth	High flux (>300 PU)	10	2.1 ± 0.4	Reference
	Mid flux (200–300 PU)	17	3.8 ± 0.6	<0.001 vs. High flux
	Low flux (180–200 PU)	4	5.9 ± 0.8	<0.001 vs. High flux (exploratory)

Comparisons: Mann–Whitney U (ICP subgroups); Kruskal–Wallis with Dunn’s post hoc (LDI subgroups). *p*-values involving Group III (n = 3) and the low-flux subgroup (n = 4) are exploratory and hypothesis-generating. EDT_50_: enzymatic decompression half-time; PU: perfusion units.

**Table 4:** Pre-specified correlations between LDI flux, ICP, and EDT_50_ (whole cohort, n = 31, Pearson r).

Correlation	r	95% CI	*p*-Value	Interpretation
Pre-Tx LDI flux vs. T0 ICP	−0.71	[−0.85, −0.49]	<0.001	Deeper burns → higher baseline ICP
Pre-Tx LDI flux vs. EDT_50_	−0.64	[−0.81, −0.39]	<0.001	Deeper burns → slower decompression kinetics
ΔFlux (T24 h−T0) vs. ΔP (T0−T24 h)	+0.58	[+0.27, +0.78]	0.001	Greater escharolysis → greater ICP reduction

LDI: Laser Doppler Imaging; ICP: intracompartmental pressure; Pre-Tx: pre-treatment; EDT_50_: enzymatic decompression half-time; ΔFlux: T24 h flux − T0 flux; ΔP: T0 ICP − T24 h ICP.

**Table 5:** Clinical and wound healing outcomes by ICP subgroup. Group III (n = 3) values are descriptive.

Outcome	Group I (n = 8)	Group II (n = 20)	Group III (n = 3)	Total (n = 31)
Clinical BICS developed	0 (0%)	0 (0%)	0 (0%)	0 (0%)
Surgical escalation triggered	0 (0%)	0 (0%)	0 (0%)	0 (0%)
ICP rebound observed	None	None	None	None
Distal perfusion maintained	8/8 (100%)	20/20 (100%)	3/3 (100%)	31/31 (100%)
P-sign count ≥3 at any point	0	0	0	0
LDI T24 h flux > 200 PU	8/8 (100%)	19/20 (95%)	1/3 (33%)	28/31 (90.3%)
Spontaneous healing (no graft)	6/8 (75%)	13/20 (65%)	1/3 (33%)	20/31 (64.5%)
Skin grafting required	2/8 (25%)	7/20 (35%)	2/3 (67%)	11/31 (35.5%)
Median wound closure (days)	14 (IQR 12–17)	17 (IQR 14–21)	23 (IQR 19–28)	17 (IQR 13–21)
Median hospital stay (days)	12 (IQR 9–15)	16 (IQR 13–22)	21 (IQR 18–26)	16 (IQR 11–21)

BICS: burn-induced compartment syndrome; LDI: Laser Doppler Imaging; PU: perfusion units; IQR: interquartile range.

**Table 6:** Exploratory, hypothesis-generating framework (not a validated clinical algorithm or practice recommendation): candidate enzymatic-debridement eligibility and monitoring hypotheses by ICP and LDI profile, proposed for prospective testing.

Clinical Scenario	ICP (mmHg)	P-Signs	Delta-P	LDI Flux (PU)	Approach Under Evaluation	Monitoring Endpoint (Hypothesized)
Established BICS	Any	≥4	—	Any	Immediate surgical escharotomy	N/A
Rapid deterioration	Rising	≥3 or new deficit	Falling	Any	Surgical escharotomy (no delay)	N/A
Grade III dominant	Any	Any	Any	<150	Surgical escharotomy	N/A
Scald mechanism, deep	20–30	1–2	≥30	<250	Surgical preferred [22]	N/A
Group II, high flux	20–30	1–2	≥40	300–400	Enzymatic debridement	Assess at T4 h (EDT_50_ ≈ 2 h)
Group II, mid flux	20–30	1–2	≥35	200–300	Enzymatic debridement	Assess at T8 h (EDT_50_ ≈ 4 h)
Group II, low flux	20–30	1–2	≥30	180–200	Enzymatic debridement + enhanced monitoring	Assess at T24 h (EDT_50_ ≈ 6 h)
Group III, strict criteria	30–40	≤2	≥30	180–300	Enzymatic debridement + enhanced monitoring [see text]	T8 h–T24 h; immediate escalation if any deterioration
Group I	<20	0–1	≥45	Any (≥180)	Enzymatic debridement	Confirm at T4 h; no escalation concern

ICP: intracompartmental pressure; EDT_50_: enzymatic decompression half-time; BICS: burn-induced compartment syndrome; LDI: Laser Doppler Imaging; PU: perfusion units. Ref. [22]: Tapking et al. Burns 2022. Group III eligibility would require simultaneous satisfaction of all listed criteria. Surgical escharotomy cannot be delayed in any case of clinical uncertainty. This table must not be used to guide clinical decisions in its current, unvalidated form.

## Data Availability

The data presented in this study are available from the corresponding author on reasonable request, subject to institutional privacy regulations.

## Abbreviations

The following abbreviations are used in this manuscript:

Abbreviation	Definition
BICS	burn-induced compartment syndrome
ICP	intra-compartmental pressure (mmHg)
EDT_50_	enzymatic decompression half-time (time to 50% of total T0 → T24 h ICP reduction)
LDI	Laser Doppler Imaging
PU	perfusion units (arbitrary, Moor LDI2-BI)
TBSA	total body surface area (%)
ABSI	Abbreviated Burn Severity Index
NexoBrid^®^	bromelain-based enzymatic debridement (MediWound Ltd.)
STIC™	Stryker Intracompartmental Pressure Monitor System
T0/T2 h/T4 h/T8 h/T24 h	ICP measurement time points
ΔP	ICP change (T0 − T24 h, mmHg)
ΔFlux	LDI flux change (T24 h − T0, PU)
Delta-P	diastolic blood pressure − ICP (mmHg)
IQR	interquartile range
SD	standard deviation
η^2^	eta-squared (ANOVA effect size)
STSG	split-thickness skin graft
dBP	diastolic blood pressure
